# Prevalence of Hypertension Among Pregnant Women When Using the 2017 American College of Cardiology/American Heart Association Blood Pressure Guidelines and Association With Maternal and Fetal Outcomes

**DOI:** 10.1001/jamanetworkopen.2021.3808

**Published:** 2021-03-31

**Authors:** Natalie A. Bello, Hui Zhou, T. Craig Cheetham, Eliza Miller, Darios T. Getahun, Michael J. Fassett, Kristi Reynolds

**Affiliations:** 1 Division of Cardiology, Department of Medicine, Columbia University Irving Medical Center, New York, New York; 2 Department of Research and Evaluation, Kaiser Permanente Southern California, Pasadena, California; 3 Department of Health Systems Science, Kaiser Permanente Bernard J. Tyson School of Medicine, Pasadena, California; 4 Department of Biomedical and Pharmaceutical Sciences, School of Pharmacy, Chapman University, Irvine, California; 5 Department of Neurology, Vagelos College of Physicians and Surgeons, Columbia University New York, New York; 6 Department of Maternal-Fetal Medicine, Kaiser Permanente West Los Angeles Medical Center, Los Angeles, California

## Abstract

**Importance:**

Hypertensive disorders of pregnancy are a leading cause of maternal morbidity and mortality. The impact of applying recent guideline definitions for nonpregnant adults to pregnant women is unclear.

**Objective:**

To determine whether reclassification of hypertensive status using the 2017 American College of Cardiology/American Heart Association (ACC/AHA) guideline definition better identifies women at risk for preeclampsia or eclampsia and adverse fetal/neonatal events compared with the current American College of Obstetricians and Gynecologists (ACOG) definition of hypertension.

**Design, Setting, and Participants:**

This cohort study used electronic medical record data of women who delivered singleton infants between 2009 and 2014 at a large US regional health system. Data analysis was performed from July 2020 to September 2020.

**Exposure:**

Application of ACC/AHA and ACOG guidelines for the definition of chronic and gestational hypertension.

**Main Outcomes and Measures:**

The primary maternal end point was the development of preeclampsia or eclampsia, and the primary fetal/neonatal end point was a composite of preterm birth, small for gestational age, and neonatal intensive care unit admission within 28 days of delivery. Net reclassification indices were calculated to examine how well the lower ACC/AHA diagnostic threshold reclassifies outcomes of pregnancy compared with the current ACOG definition of hypertension.

**Results:**

Applying the ACC/AHA criteria to 137 389 pregnancies of women (mean [SD] age at time of delivery, 30.1 [5.8] years) resulted in a 14.3% prevalence of chronic hypertension (19 621 pregnancies) and a 13.8% prevalence of gestational hypertension (18 998 pregnancies). A 17.8% absolute increase was found in the overall prevalence of hypertension from 10.3% to 28.1%. The 2.1% of women who were reclassified with chronic rather than gestational hypertension had the highest risk of developing preeclampsia compared with women without hypertension by either criterion (adjusted risk ratio, 13.58; 95% CI, 12.49-14.77). Overall, the use of the ACC/AHA criteria to diagnose hypertension resulted in a 20.8% improvement in the appropriate identification of future preeclampsia, but only a 3.8% improvement of appropriate fetal/neonatal risk classification.

**Conclusions and Relevance:**

Using the lower diagnostic threshold for hypertension recommended in the 2017 ACC/AHA guideline increased the prevalence of chronic and gestational hypertension, markedly improved the appropriate identification of women who would go on to develop preeclampsia, and was associated with the identification of adverse fetal/neonatal risk.

## Key Points

**Question:**

How does the prevalence of hypertension in pregnant women change when using the 2017 American College of Cardiology/American Heart Association definition, which is lower than the American College of Obstetricians and Gynecologists threshold, and is there an association with maternal or fetal outcomes?

**Findings:**

In this cohort study that included 137 389 pregnancies, the prevalence of hypertension increased from 10.3% to 28.1% and resulted in a net reclassification index of 20.8% for the identification of future preeclampsia and 3.8% for the identification of fetal/neonatal adverse events.

**Meaning:**

These findings suggest that applying the lower diagnostic thresholds to pregnant women may better identify women at risk of adverse events.

## Teaser

This cohort study estimates the prevalence of hypertension among pregnant women using the 2017 American College of Cardiology/American Heart Association (ACC/AHA) definition of hypertension instead of the American College of Obstetricians and Gynecologists threshold and assesses identification of adverse maternal or fetal outcomes associated with use of the ACC/AHA guidelines.

## Introduction

Hypertension is the most common medical comorbidity encountered during pregnancy,^1^ and hypertensive disorders of pregnancy are associated with an increased risk of adverse outcomes for both mother and fetus.^2,3^ In 2017, the American College of Cardiology and American Heart Association (ACC/AHA) released an updated clinical practice guideline for the prevention, detection, evaluation, and management of high blood pressure in adults.^4^ This guideline changed the criteria for diagnosing hypertension in nonpregnant adults to a blood pressure (BP) of 130/80 mm Hg or higher, lower than the previous cutoff of 140/90 mm Hg or higher. The American College of Obstetricians and Gynecologists (ACOG) did not adopt the lower threshold and continues to define hypertension in pregnancy as 140/90 mm Hg or higher.^2^ Several prior studies have reported that, as expected, the application of lower diagnostic thresholds for hypertension is associated with an increased prevalence of chronic hypertension (between 8.5% and 10.0% higher).^5,6,7^ To our knowledge, this is the first US-based study to examine the application of the lower threshold to women throughout the duration of pregnancy, rather than only prior to 20 weeks’ gestation.^8^

The objectives of this investigation were to estimate changes in diagnostic information that may result from applying the 2017 ACC/AHA BP guideline criteria to pregnant women and their offspring. The first goal was to establish the impact of using this standard on the prevalence of chronic and gestational hypertension in pregnancy. The second goal was to quantify how reclassification was associated with estimates of maternal and fetal/neonatal outcomes, including preeclampsia and eclampsia, preterm birth, small for gestational age, and admission to a neonatal intensive care unit (NICU) within 28 days of delivery.

## Methods

This project was approved by the Kaiser Permanente institutional review board, which granted a waiver of informed consent and authorization under the Health Insurance Portability and Accountability Act. We followed the Strengthening the Reporting of Observational Studies in Epidemiology (STROBE) reporting guideline for cohort studies.

### Study Population

This study included a birth cohort of all singleton deliveries at Kaiser Permanente Southern California (KPSC) between January 1, 2009, and December 31, 2014, among women aged 15 to 49 years on the delivery date. KPSC is an integrated health care system that provides medical services to 4.7 million members. The membership is racially and socioeconomically diverse and is representative of the population of Southern California.^9^ Rigorous office BP measurements became a routine part of the KPSC electronic medical record in 2008, and the study start date was chosen to allow an examination of women with documented office BP measurements for the 6 months prior to the start of pregnancy through delivery. Women were required to have continuous membership in the health care system from 6 months prior to the start of pregnancy to the delivery date (allowing an administrative gap of 60 days in membership), and to deliver at a viable gestational age between 22 and 43 weeks to be eligible for inclusion. An estimated delivery date was calculated based on self-reported last menstrual period with first trimester ultrasonographic confirmation. Using the estimated delivery date, the start of pregnancy was calculated by subtracting 280 days. Women were excluded if gestational age could not be ascertained (178 women) or if office BP was not measured prior to 20 weeks’ gestation, unless the patient was prescribed antihypertensive medication(s) (1571 patients). Women were also excluded if they had a medical comorbidity for which antihypertensive medications might have been used for an indication other than lowering BP (113 women), or if they were prescribed teratogenic medications from 6 months prior to pregnancy through delivery (3975 women); see eTable 1 in the Supplement for a listing of exclusion diagnoses and exclusion teratogenic medications. The final analytic sample included 137 389 unique pregnancies (eFigure 1 in the Supplement).

Maternal and infant social and demographic characteristics, medical and pregnancy history, and maternal vital signs and body mass index (BMI; calculated as weight in kilograms divided by height in meters squared) were extracted from the KPSC Perinatal Data Mart, which compiles information from the Perinatal Service System, Automated Vital Statistics System, and the electronic medical record.^10^ Race and ethnicity were self-reported and are presented to demonstrate the generalizability of these findings to a diverse population.

### Definitions of Hypertension

**Figure 1.**
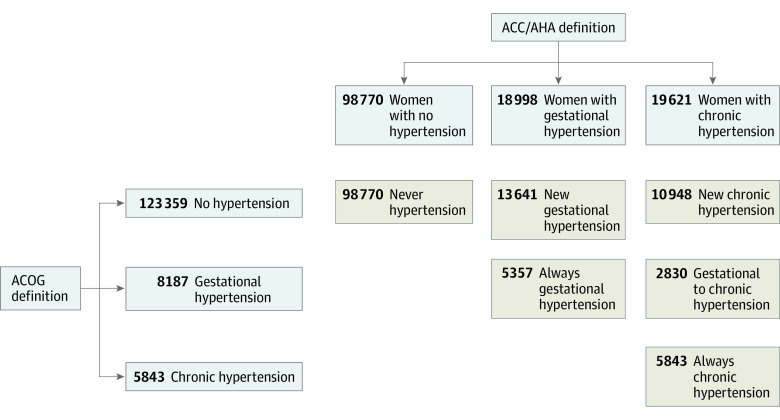
Cross-Classification of Women From ACOG to ACC/AHA Category Resulting in 6 Possible Groups ACOG indicates American College of Obstetrics and Gynecology; ACC/AHA, American College of Cardiology/American Heart Association. Study terms for reclassification include: new chronic hypertension (no ACOG hypertension and ACC/AHA chronic hypertension), gestational to chronic hypertension (ACOG gestational hypertension and ACC/AHA chronic hypertension), new gestational hypertension (no ACOG hypertension, ACC/AHA gestational hypertension), always chronic hypertension (ACOG and ACC/AHA chronic hypertension), always gestational hypertension (ACOG and ACC/AHA gestational hypertension), and never hypertension (no ACOG or ACC/AHA hypertension).

The criteria used to determine outpatient BP and hypertension diagnosis in this cohort have been previously described.^11^ Briefly, hypertension was defined as any of the following: (1) office BP meeting or exceeding the ACOG or ACC/AHA diagnostic threshold on 2 or more days within 30 days of one another; (2) an *International Classification of Diseases, Ninth Revision *(*ICD-9*) or *International Statistical Classification of Diseases and Related Health Problems, Tenth Revision *(*ICD-10*) diagnosis code for hypertension (eTable 2 in the Supplement) and at least 1 prescription fill for an antihypertensive medication; (3) at least 1 office BP measurement meeting or exceeding the diagnostic threshold and at least 1 prescription fill for an antihypertensive medication; or (4) at least 1 office BP measurement meeting or exceeding the diagnostic threshold and an *ICD-9* or *ICD-10* diagnosis code for hypertension (eTable1 in the Supplement). The ACOG diagnostic threshold was defined as an office systolic BP (SBP) of 140 mm Hg or higher and/or a diastolic BP (DBP) of 90 mm Hg or higher, and the ACC/AHA diagnostic threshold was defined as an office SBP of 130 mm Hg or higher and/or a DBP of 80 mm Hg or higher. Chronic and gestational hypertension were defined according to standard definitions.^2^ Hypertension that predated pregnancy or occurred prior to 20 weeks’ gestation was classified as chronic hypertension, and hypertension that occurred at 20 weeks’ gestation or later was classified as gestational hypertension. In instances when the diagnostic criteria spanned 20 weeks of gestation (ie, an office BP that met or exceeded the diagnostic threshold occurred at 18 weeks, but the prescription fill occurred at 21 weeks), the case was classified as chronic hypertension if the second qualifying event occurred within 6 weeks of the initial criteria being met. All women were categorized as having no hypertension, chronic hypertension, or gestational hypertension by both the ACOG and the ACC/AHA criteria. Finally, women were cross-classified by their ACOG and ACC/AHA group into 1 of 6 categories describing the change, if any, from ACOG to ACC/AHA criteria: new chronic hypertension (ie, ACOG no hypertension and ACC/AHA chronic hypertension), gestational to chronic hypertension (ACOG gestational hypertension and ACC/AHA chronic hypertension), new gestational hypertension (ACOG no hypertension, ACC/AHA gestational hypertension), always chronic hypertension (ACOG and ACC/AHA chronic hypertension), always gestational hypertension (ACOG and ACC/AHA gestational hypertension), and never hypertension (no ACOG or ACC/AHA hypertension) (Figure 1).

### Outcomes

The primary maternal outcome was the development of preeclampsia or eclampsia, defined using *ICD-9* and *ICD-10* diagnosis codes (eTable2 in the Supplement). The primary fetal/neonatal outcome was a composite of preterm birth (defined as a delivery prior to 37 weeks of gestational age), small for gestational age (defined as a birth weight ≤10th percentile based on gender, race, and gestational age using the Okin criteria^12^), and NICU admission during the first 28 days of life. In secondary analyses, each component of the primary fetal/neonatal outcome was examined separately. Analyses of preterm birth in women with gestational hypertension were restricted to the subset of women who were diagnosed with gestational hypertension prior to 37 weeks (9458 women total, 5755 with new gestational hypertension and 3703 with always gestational hypertension).

### Statistical Analysis

Descriptive analysis was performed to compare maternal characteristics among the 6 ACOG vs ACC/AHA cross-classification groups. Continuous variables are presented as mean values (with SD) and categorical variables are presented as frequencies (ie, percentages). Mean SBP and DBP were defined using all available clinic values for each trimester. Occurrences of maternal and fetal/neonatal outcomes were calculated per 1000 singleton live births for each of the 6 cross-classification groups. Because the majority of outcome occurrences were greater than 10%, risk ratios and a corresponding 95% confidence interval for each outcome were calculated using Poisson models with robust error variance to compare each of the 5 hypertensive cross-classified groups to the never hypertensive referent group before and after adjustment for potential confounders.^13^ For all analyses involving preterm birth, the risk set of women only included those who delivered before 37 weeks’ gestation; all other analyses were performed using data from the entire cohort. All models were a priori adjusted for potential confounders including maternal age, maternal race/ethnicity, early pregnancy BMI, smoking during pregnancy, preexisting and gestational diabetes, and parity.

Net reclassification indices (NRI) were calculated to evaluate for an improvement in prediction of adverse maternal and fetal/neonatal outcomes using the lower ACC/AHA diagnostic threshold for hypertension for pregnant women in place of the higher ACOG threshold.^14^ Specifically, we followed 5 steps: (1) for each outcome, we defined those who developed the outcome of interest as “events” and those without the outcome as “nonevents”; (2) for each outcome, chronic hypertension was considered the highest risk group, gestational hypertension was considered an intermediate risk group, and no hypertension was considered the group with lowest risk; (3) the ACOG criteria was considered the old guideline and the ACC/AHA criteria was considered the new guideline; (4) any movement from a lower risk group in the old guideline to a higher risk group in new guideline was considered “up” and any movement from a higher risk group in the old guideline to a lower risk group in the new guideline was considered “down”; and (5) using the formula NRI = (*P*{up event} − *P*{down event}) + (*P*{down nonevent} − *P*{up nonevent}), we calculated the percentage of up and down difference in event (sensitivity increase as *P*{up event} − *P*{down event}) and nonevent (specificity increase as *P*{down nonevent} − *P*{up nonevent}) between the “old” ACOG and “new” ACC/AHA criteria. The NRI is the sum of the sensitivity and specificity increases.

Statistical analyses were conducted using SAS Enterprise Guide 5.1 (SAS Institute) from July to September 2020. All hypothesis tests were 2-sided with a significance level of *P* < .05.

## Results

**Figure 2.**
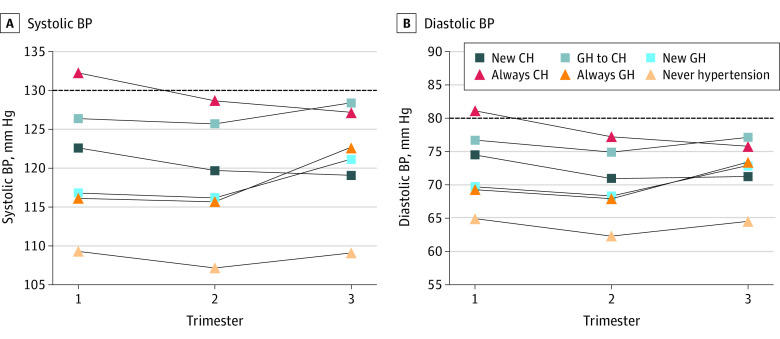
Mean Systolic and Diastolic Blood Pressure (BP) by Trimester According to Cross-Classification Status The dashed line indicates ACC/AHA diagnostic threshold for systolic (130 mm Hg) and diastolic hypertension (80 mm Hg). ACOG indicates American College of Obstetrics and Gynecology; ACC/AHA, American College of Cardiology/American Heart Association; CH, chronic hypertension; GH, gestational hypertension. Study terms for reclassification include: new chronic hypertension (no ACOG hypertension and ACC/AHA chronic hypertension), gestational to chronic hypertension (ACOG gestational hypertension and ACC/AHA chronic hypertension), new gestational hypertension (no ACOG hypertension, ACC/AHA gestational hypertension), always chronic hypertension (ACOG and ACC/AHA chronic hypertension), always gestational hypertension (ACOG and ACC/AHA gestational hypertension), and never hypertension (no ACOG or ACC/AHA hypertension).

Of the 137 389 women included for analysis, the mean (SD) age at the time of delivery was 30.1 (5.8) years, mean (SD) early pregnancy BMI was 27.0 (6.3), 1775 women (1.3%) had preexisting diabetes, and 20 497 (14.9%) experienced gestational diabetes (Table 1). By race/ethnicity, the cohort consisted of 35 014 (25.5%) White non-Hispanic women, 70 840 (51.6%) Hispanic women, 11 251 (8.2%) Black non-Hispanic women, and 17 838 (13.0%) women who were Asian/Pacific Islander. This was the first pregnancy for 55 845 women (40.6%). As depicted in Figure 1, using ACOG criteria, 5843 of women (4.3%) had chronic hypertension and 8187 (6.0%) had gestational hypertension. When the lower ACC/AHA diagnostic threshold for hypertension was applied, 19 621 women (14.3%) had chronic hypertension and 18 998 (13.8%) had gestational hypertension. The application of the ACC/AHA lower diagnostic criteria for hypertension resulted in the reclassification of hypertensive status for 27 419 women (20.0%); 10 948 (8.0%) were newly diagnosed with chronic hypertension, 13 641 (9.9%) were newly diagnosed with gestational hypertension, and 2830 (2.1%) were reclassified with chronic rather than gestational hypertension. Figure 2 depicts mean BP by trimester for women in each of the 6 ACOG–ACC/AHA cross-classification groups.

**Table 1.** Maternal Characteristics at the Time of Index Pregnancy Overall, and by Change From ACOG to ACC/AHA BP Categories

Characteristic	Women, No. (%)						
	Overall (N = 137 389)	Hypertension^a^					
		New chronic (n = 10 948)	Gestational to chronic (n = 2830)	New gestational (n = 13 641)	Always chronic (n = 5843)	Always gestational (n = 5357)	Never (n = 98 770)
Age at time of delivery, mean (SD), y	30.1 (5.8)	30.7 (5.6)	30.8 (5.7)	29.6 (5.9)	32.9 (5.4)	29.9 (6.2)	29.9 (5.8)
Race/ethnicity							
White	35 014 (25.5)	3031 (27.7)	918 (32.4)	4526 (33.2)	1506 (25.8)	1589 (29.7)	23 444 (23.7)
Asian	17 838 (13.0)	1006 (9.2)	272 (9.6)	1377 (10.1)	679 (11.6)	588 (11.0)	13 916 (14.1)
Black	11 251 (8.2)	1160 (10.6)	342 (12.1)	1028 (7.5)	836 (14.3)	583 (10.9)	7302 (7.4)
Hispanic	70 840 (51.6)	5542 (50.6)	1246 (44.0)	6434 (47.2)	2676 (45.8)	2501 (46.7)	52 441 (53.1)
Other/unknown	2446 (1.8)	209 (1.9)	52 (1.8)	276 (2.0)	146 (2.5)	96 (1.8)	1667 (1.7)
Annual family income, $							
<50 000	48 698 (35.4)	3971 (36.3)	1017 (35.9)	4693 (34.4)	2110 (36.1)	2039 (38.1)	34 868 (35.3)
50 000-99 999	77 097 (56.1)	6175 (56.4)	1616 (57.1)	7817 (57.3)	3324 (56.9)	2885 (53.9)	55 280 (56.0)
≥100 000	11 425 (8.3)	791 (7.2)	195 (6.9)	1112 (8.2)	403 (6.9)	426 (8.0)	8498 (8.6)
Unknown	169 (0.1)	11 (0.1)	2 (0.1)	19 (0.1)	6 (0.1)	7 (0.1)	124 (0.1)
Education							
High school or less	43 059 (31.3)	3339 (30.5)	838 (29.6)	4296 (31.5)	1702 (29.1)	1667 (31.1)	31 217 (31.6)
College	73 817 (53.7)	6124 (55.9)	1580 (55.8)	7411 (54.3)	3317 (56.8)	2926 (54.6)	52 459 (53.1)
Graduate	20 305 (14.8)	1474 (13.5)	409 (14.5)	1918 (14.1)	821 (14.1)	753 (14.1)	14 930 (15.1)
Unknown	208 (0.2)	11 (0.1)	3 (0.1)	16 (0.1)	3 (0.1)	11 (0.2)	164 (0.2)
Speaking language other than English at home	10 489 (7.6)	569 (5.2)	116 (4.1)	751 (5.5)	295 (5.0)	275 (5.1)	8483 (8.6)
Marital status							
Single	31 048 (22.6)	2398 (21.9)	627 (22.2)	3286 (24.1)	1139 (19.5)	1411 (26.3)	22 187 (22.5)
Married	101 218 (73.7)	8075 (73.8)	2071 (73.2)	9845 (72.2)	4421 (75.7)	3732 (69.7)	73 074 (74.0)
Separated/divorced	3386 (2.5)	333 (3.0)	97 (3.4)	344 (2.5)	206 (3.5)	151 (2.8)	2255 (2.3)
Unknown	1737 (1.3)	142 (1.3)	35 (1.2)	166 (1.2)	77 (1.3)	63 (1.2)	1254 (1.3)
Early pregnancy BMI, mean (SD)^b^	27.0 (6.3)	30.5 (7.1)	32.2 (7.9)	28.3 (6.5)	33.1 (8.0)	28.1 (6.6)	25.8 (5.5)
Preexisting diabetes (type 1 or 2)	1775 (1.3)	302 (2.8)	95 (3.4)	179 (1.3)	516 (8.8)	117 (2.2)	566 (0.6)
Gestational diabetes	20 497 (14.9)	2414 (22.0)	704 (24.9)	2103 (15.4)	1787 (30.6)	979 (18.3)	12 510 (12.7)
Cigarette smoking^c^	2166 (1.6)	170 (1.6)	70 (2.5)	306 (2.2)	99 (1.7)	109 (2)	1412 (1.4)
Parity							
0	55 845 (40.6)	4604 (42.1)	1298 (45.9)	6121 (44.9)	2428 (41.6)	2375 (44.3)	39 019 (39.5)
1	43 090 (31.4)	3432 (31.3)	795 (28.1)	4003 (29.3)	1759 (30.1)	1536 (28.7)	31 565 (32.0)
≥2	27 638 (20.1)	2142 (19.6)	515 (18.2)	2241 (16.4)	1316 (22.5)	963 (18.0)	20 461 (20.7)
Unknown	10 816 (7.9)	770 (7.0)	222 (7.8)	1276 (9.4)	340 (5.8)	483 (9.0)	7725 (7.8)
Girls	67 057 (48.8)	5289 (48.3)	1325 (46.8)	6586 (48.3)	2855 (48.9)	2544 (47.5)	48 458 (49.1)
Cesarean birth	41 123 (29.9)	3907 (35.7)	1187 (41.9)	4475 (32.8)	2849 (48.8)	1962 (36.6)	26 743 (27.1)

Abbreviations: ACOG, American College of Obstetrics and Gynecology; ACC/AHA, American College of Cardiology/American Heart Association; BMI, body mass index.

^a^ Study terms for reclassification include: new chronic hypertension (no ACOG hypertension and ACC/AHA chronic hypertension), gestational to chronic hypertension (ACOG gestational hypertension and ACC/AHA chronic hypertension), new gestational hypertension (no ACOG hypertension, ACC/AHA gestational hypertension), always chronic hypertension (ACOG and ACC/AHA chronic hypertension), always gestational hypertension (ACOG and ACC/AHA gestational hypertension), and never hypertension (no ACOG or ACC/AHA hypertension).

^b^ Calculated as weight in kilograms divided by height in meters squared.

^c^ Cigarette smoking from 3 months prior to pregnancy through delivery.

### Maternal and Fetal/Neonatal Outcomes

**Figure 3.**
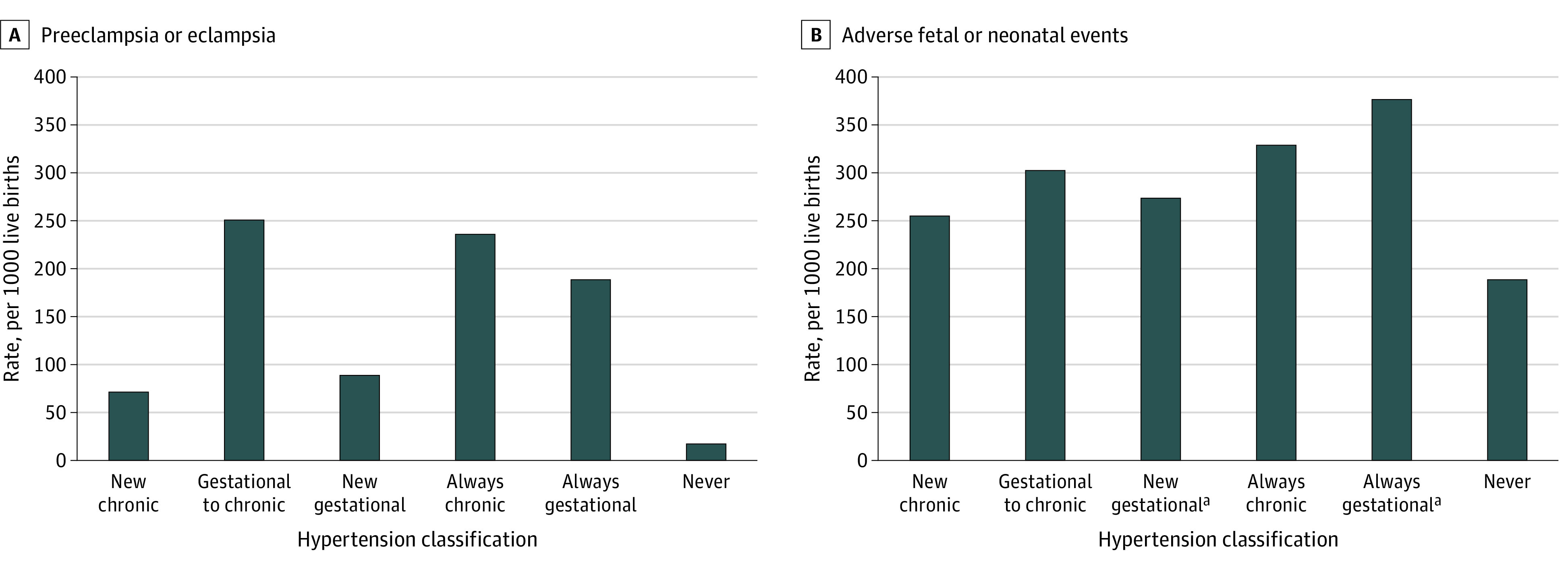
Rates of Preeclampsia/Eclampsia and Adverse Fetal/Neonatal Events by Hypertension Classification ACOG indicates American College of Obstetrics and Gynecology; ACC/AHA, American College of Cardiology/American Heart Association. Study terms for reclassification include: new chronic hypertension (no ACOG hypertension and ACC/AHA chronic hypertension), gestational to chronic hypertension (ACOG gestational hypertension and ACC/AHA chronic hypertension), new gestational hypertension (no ACOG hypertension, ACC/AHA gestational hypertension), always chronic hypertension (ACOG and ACC/AHA chronic hypertension), always gestational hypertension (ACOG and ACC/AHA gestational hypertension), and never hypertension (no ACOG or ACC/AHA hypertension). ^a^For analyses involving preterm birth, women who were diagnosed with gestational hypertension after 37 weeks were excluded from these analyses (new gestational hypertension [5755 women] and always gestational hypertension [3703 women]).

Compared with women who were never hypertensive, women with ACOG gestational hypertension who were reclassified with chronic hypertension using ACC/AHA criteria had the highest rate of preeclampsia/eclampsia (adjusted relative risk [RR], 13.58; 95% CI, 12.49-14.77) (Figure 3 and Table 2). Women with chronic hypertension by both criteria had the next highest rate of preeclampsia/eclampsia (adjusted RR, 12.54; 95% CI, 11.65-13.49) followed by women with gestational hypertension by both criteria (adjusted RR, 10.54; 95% CI, 9.78-11.35). Women newly diagnosed with gestational and chronic hypertension by ACC/AHA criteria also had statistically significantly higher rates of preeclampsia/eclampsia than never hypertensive women, which persisted after adjustment (new gestational: adjusted RR, 5.11; 95% CI, 4.74-5.50; new chronic: adjusted RR, 3.92; 95% CI, 3.60-4.26). Using the ACC/AHA guideline to reclassify women with hypertension at a lower BP threshold resulted in an NRI-assessed 20.8% improvement in the appropriate identification of women with preeclampsia.

**Table 2.** Adverse Maternal and Fetal/Neonatal Outcomes With ACOG vs ACC/AHA Blood Pressure (BP) Classification of Hypertensive Disorders of Pregnancy

Adverse outcome	Relative risk (95% CI)									
	New chronic hypertension^a^		Gestational to chronic hypertension^a^		New gestational hypertension^a^		Always chronic hypertension^a^		Always gestational hypertension^a^	
	Unadjusted	Adjusted^b^	Unadjusted	Adjusted^b^	Unadjusted	Adjusted^b^	Unadjusted	Adjusted^b^	Unadjusted	Adjusted^b^
Preeclampsia/eclampsia	4.12 (3.79-4.48)	3.92 (3.60-4.26)	14.49 (13.38-15.70)	13.58 (12.49-14.77)	5.18 (4.82-5.57)	5.11 (4.74-5.50)	13.63 (12.75-14.57)	12.54 (11.65-13.49)	10.90 (10.12-11.74)	10.54 (9.78-11.35)
Fetal/neonatal composite outcome^c^	1.20 (1.16-1.25)	1.24 (1.20-1.29)	1.61 (1.52-1.71)	1.68 (1.58-1.78)	1.46 (1.39-1.52)	1.49 (1.42-1.56)	1.74 (1.67-1.81)	1.80 (1.72-1.87)	2.00 (1.91-2.09)	2.00 (1.92-2.10)
Preterm birth (<37 wk)^c^	1.51 (1.42-1.60)	1.48 (1.39-1.57)	2.27 (2.07-2.48)	2.21 (2.02-2.43)	1.73 (1.60-1.87)	1.73 (1.60-1.87)	2.80 (2.64-2.97)	2.59 (2.43-2.76)	3.47 (3.26-3.70)	3.38 (3.17-3.61)
Small for gestational age	1.01 (0.95-1.08)	1.23 (1.15-1.31)	1.38 (1.24-1.52)	1.77 (1.60-1.96)	1.17 (1.11-1.23)	1.29 (1.22-1.37)	1.36 (1.26-1.46)	1.83 (1.69-1.97)	1.50 (1.40-1.62)	1.64 (1.53-1.77)
NICU admission	1.36 (1.28-1.45)	1.24 (1.17-1.32)	1.90 (1.72-2.08)	1.65 (1.50-1.82)	1.25 (1.17-1.32)	1.18 (1.11-1.25)	2.10 (1.97-2.24)	1.76 (1.65-1.89)	2.04 (1.91-2.19)	1.90 (1.78-2.04)

Abbreviations: ACC/AHA, American College of Cardiology/American Heart Association; ACOG, American College of Obstetricians and Gynecologists; BMI, body mass index; NICU, neonatal intensive care unit.

^a^ Study terms for reclassification include: new chronic hypertension (no ACOG hypertension and ACC/AHA chronic hypertension), gestational to chronic hypertension (ACOG gestational hypertension and ACC/AHA chronic hypertension), new gestational hypertension (no ACOG hypertension, ACC/AHA gestational hypertension), always chronic hypertension (ACOG and ACC/AHA chronic hypertension), always gestational hypertension (ACOG and ACC/AHA gestational hypertension).

^b^ Adjusted for maternal age, race/ethnicity, BMI at early pregnancy, smoking, diabetes (preexisting and gestational diabetes), and parity.

^c^ Excludes 9540 women with gestational hypertension diagnosed after 37 weeks.

Women with gestational hypertension by both ACOG and ACC/AHA criteria experienced the highest rates of adverse fetal/neonatal events (Figure 3, Table 2; eFigure 3 in the Supplement). The increased event rate in this group was predominantly driven by a greater number of preterm births compared with never hypertensive women (adjusted RR, 3.38; 95% CI, 3.17-3.61). The use of lower BP thresholds improved the estimation of risk of an adverse fetal/neonatal outcome by 3.8% for the composite end point, ranging from an NRI of 1.4% for small for gestational age to 5.5% for preterm birth.

## Discussion

In this retrospective cohort study examining women who received medical care through an integrated health system and gave birth to a singleton infant, the reclassification of hypertensive status using the 2017 ACC/AHA criteria rather than the ACOG recommendations resulted in a 17.8% increase in the prevalence of any hypertensive disorder. Reclassification, which included new diagnoses of chronic and gestational hypertension for some women and the transition from a diagnosis of gestational to chronic hypertension in others, resulted in a 20.8% improvement in the appropriate identification of women at risk for preeclampsia. The highest level of maternal risk for preeclampsia was seen in the group of women reclassified with chronic hypertension by ACC/AHA criteria who are currently diagnosed with gestational hypertension using ACOG criteria. Despite having a marked impact on the identification of high-risk women, the change in classification had a lesser impact on the identification of neonates at risk for adverse events defined using the NRI.

In 2017, the ACC/AHA lowered the diagnostic threshold for hypertension to a BP of 130/80 mm Hg or higher based on outcomes data from randomized trials of BP lowering in nonpregnant adults.^4^ In the absence of similar data from pregnant women, ACOG continues to define hypertension as a BP of 140/90 mm Hg or higher, and recommends blood pressure medication initiation once SBP is 160 mm Hg and/or DBP is 110 mm Hg. We found a 10 mm Hg decrease in the SBP and DBP diagnostic thresholds for hypertension resulted in a 10.0% absolute increase in the prevalence of chronic hypertension during pregnancy. This finding is consistent with 3 prior US-based studies that have examined the impact of applying the ACC/AHA diagnostic criteria and reported increased prevalence of chronic hypertension ranging from 8.5% to 10.0%.^5,6,7^ To our knowledge there has only been a single prior study examining the impact of applying the 2017 ACC/AHA on the prevalence of gestational hypertension, which drew its data from a group of 16 345 women from China.^8^ In that study, the authors reported a 20.9% increase in the prevalence of gestational hypertension defined using the 2017 ACC/AHA criteria compared with the 7.8% increase in the current study. Although the same diagnostic threshold for hypertension was used in both analyses, Hu et al^8^ diagnosed gestational hypertension using a single office BP measurement. Our study followed the diagnostic recommendations of the 2017 ACC/AHA guideline and required a minimum of 2 office BP measures of 130/80 mm Hg or higher to diagnose both chronic and gestational hypertension. Additionally, in contrast to the rigorous BP measurement protocols in place at KPSC, office BP was not measured in a standardized fashion in the prior study, allowing for an increase in measurement error. These methodological differences may have resulted in a falsely high prevalence of gestational hypertension in the study by Hu et al^8^ because of the potential inclusion of women with an isolated elevation in blood pressure from a temporary cause such as pain, stress, or measurement error.

Prior studies found elevated BP using ACC/AHA thresholds was associated with abnormalities in hepatic, renal, and coagulation function, but excluded women with chronic hypertension and preeclampsia, thus limiting their ability to examine changes in hard clinical end points.^6^ Several groups have examined the application of the 2017 ACC/AHA diagnostic thresholds early in pregnancy and found that stage 1 hypertension was associated with an increased risk of hypertensive disorders of pregnancy including preeclampsia and gestational hypertension and adverse perinatal outcomes compared with normotension.^15,16,17,18^ Our findings confirm and extend this prior work to examine the impact of reclassifying women using not only early pregnancy BP but also outpatient BP measurements prior to pregnancy and throughout gestation up until delivery. Our approach thus mimics clinical practice. Our methodology also took into account the prescription of antihypertensive medications in addition to ICD codes and clinic BP values, which has been shown to be superior for the classification of hypertension in an outpatient population of pregnant women.^11^

In addition to increasing the prevalence of hypertension among women of childbearing age, the application of the 2017 ACC/AHA thresholds has several implications for the care of pregnant women. First, although a new diagnosis of chronic hypertension in a pregnant woman is not necessarily an indication for medication initiation, it is considered a high-level risk factor for the development of preeclampsia and is an indication for the initiation of low-dose aspirin prophylaxis according to the US Preventive Services Task Force.^19^ In this cohort, we found that women with chronic hypertension diagnosed using the ACC/AHA criteria who were previously classified as having gestational hypertension had a risk of preeclampsia that was even higher than that of women who were diagnosed with chronic hypertension using the ACOG criteria. Thus, there is a pool of women for whom reclassification with chronic hypertension and the initiation of aspirin as a preventative therapy to reduce risk of preeclampsia has the potential to reduce maternal and perinatal morbidity and mortality, which has been suggested by secondary analyses of high-risk aspirin trials.^20,21^ Further prospective trials are needed to confirm this finding. Second, pregnant women with chronic and gestational hypertension are at increased risk for adverse maternal and fetal/neonatal events and are often followed more closely during their pregnancies. The appropriate identification of women at heightened risk will help to intensify clinical surveillance in previously overlooked women and may help identify and prevent impending adverse events. Third, reclassification of women also resulted in the identification of more women with gestational diabetes and could be used to target women for educational programs during pregnancy aimed at cardiovascular lifestyle modification to reduce future risk.

### Strengths and Limitations

There are several strengths of this study. To our knowledge, this is the largest and most ethnically diverse US cohort of singleton births used to examine the implementation of the 2017 ACC/AHA definition of hypertension and changes in the prevalence of both chronic and gestational hypertension as well as the impact of reclassification on maternal and fetal/neonatal outcomes. The criteria used to determine outpatient hypertension are rigorous and well-vetted, and the KPSC health care system has a robust BP measurement methodology in place resulting in reliable office BP measures. In addition, primary end points are clinically relevant leading causes of maternal and fetal/neonatal morbidity and mortality. The primary maternal end point was based on *ICD-9* and *ICD-10* codes rather than clinical adjudication of cases and thus we did not try to further subcategorize preeclampsia as severe or not. Although this is a limitation, we believe ICD codes likely have a high specificity but unclear sensitivity, which would have biased our results toward the null. In contrast to previous analyses, when calculating risk of preterm birth in hypertensive compared with normotensive women, we only included women who gave birth prior to 37 weeks, thus avoiding an immortal time bias.

This study has several limitations that must be taken into consideration. First, we did not examine antihypertensive medication use prior to or during pregnancy in the current study. Thus, we cannot determine the effect of BP lowering and achieved BP in hypertensive women on maternal and fetal/neonatal outcomes. The results of future randomized trials of BP lowering will be needed to examine this phenomenon. Second, as this cohort included only live births, we did not examine the rate of stillbirth. Third, we have no information on maternal aspirin use during pregnancy because the use of over-the-counter medications such as low-dose aspirin are not reliably recorded in the electronic medical record. However, the lack of adjustment for the use of a medication that reduces preeclampsia risk advised only for women based on the ACOG hypertension criteria would have biased our results toward the null (against improvement using the lower BP criteria).

## Conclusions

In this cohort study, applying the lower ACC/AHA BP criteria to pregnant women resulted in a 17.8% increase in the prevalence of hypertension, with slightly more women diagnosed with chronic (14.3%) compared with gestational (13.8%) hypertension. Reclassification also markedly improved the appropriate identification of maternal risk for preeclampsia and, to a lesser extent, identified women with heightened fetal/neonatal risk as well.

## Notes

Supplement. **eTable 1.** Exclusion Lists: Teratogenic Medications, *ICD-9* Diagnostic Codes **eTable 2.** *ICD-9* Codes for Hypertension **eFigure 1.** Cohort Inclusion/Exclusion Flow Diagram **eFigure 2.** Fetal/Neonatal Outcomes: Preterm Birth, Small for Gestational Age (SGA), Neonatal Intensive Care Unit (NICU) Admission
