# Error in the Byline

**DOI:** 10.1001/jamanetworkopen.2021.12000

**Published:** 2021-04-28

In the Original Investigation titled “Prevalence of Hypertension Among Pregnant Women When Using the 2017 American College of Cardiology/American Heart Association Blood Pressure Guidelines and Association With Maternal and Fetal Outcomes,”^1^ published March 31, 2021, there was an error in the initials of an author name in the byline. The author listed should have been Darios Getahun. This article has been corrected.
