# Supplementary material for: Prevalence of Hypertension Among Pregnant Women When Using the 2017 American College of Cardiology/American Heart Association Blood Pressure Guidelines and Association With Maternal and Fetal Outcomes
Source: JAMA Netw Open. 2021 Mar 31;4(3):e213808. doi: 10.1001/jamanetworkopen.2021.3808 (PMC8013820; doi:10.1001/jamanetworkopen.2021.3808)
Supplement: Supplement. — eTable 1. Exclusion Lists: Teratogenic Medications, ICD-9 Diagnostic Codes eTable 2. ICD-9 Codes for Hypertension eFigure 1. Cohort Inclusion/Exclusion Flow Diagram eFigure 2. Fetal/Neonatal Outcomes: Preterm Birth, Small for Gestational Age (SGA), Neonatal Intensive Care Unit (NICU) Admission [file jamanetwopen-e213808-s001.pdf]

Supplemental Online Content

Bello NA, Zhou H, Cheetham TC, et al. Prevalence of hypertension among pregnant women when using the 2017 American College of Cardiology/American Heart Association blood pressure guidelines and association with maternal and fetal outcomes. *JAMA Netw Open*. 2021;4(3):e213808. doi:10.1001/jamanetworkopen.2021.3808

eTable 1. Exclusion Lists: Teratogenic Medications, *ICD-9* Diagnostic Codes
eTable 2. *ICD-9* Codes for Hypertension
eFigure 1. Cohort Inclusion/Exclusion Flow Diagram
eFigure 2. Fetal/Neonatal Outcomes: Preterm Birth, Small for Gestational Age (SGA), Neonatal Intensive Care Unit (NICU) Admission

This supplemental material has been provided by the authors to give readers additional information about their work.

© 2021 Bello NA et al. *JAMA Network Open.*

eTable 1 Exclusion lists: Teratogenic Medications, *ICD-9* Diagnostic Codes

Teratogenic Drugs

Drug	Drug Class	Exclusion
Isotretinoin	Vitamin A Analog	Yes
Bexarotene	Vitamin A Analog	Yes
Acitretin	Vitamin A Analog	Yes
Misoprostol	Prostaglandin Analog	Yes
Methotrexate	Antineoplastic	Yes
Mycophenolate	Immunosuppressant	Yes
Thalidomide	Immunologic	Yes
Warfarin	Anticoagulant	Yes
Lithium	Mood Stabilizer	Yes
Azathioprine	Immunologic	Yes
Leflunomide	Anti-rheumatic	Yes
Valproic Acid<br>Sodium Valproate<br>Divalproex	Anticonvulsant	Yes
Carbamazepine	Anticonvulsant	Yes
Phenytoin (Fosphenytoin)	Anticonvulsant	Yes
Phenobarbital (Mephobarbital)	Anticonvulsant	Yes
Primidone	Anticonvulsant	Yes
Ethosuximide	Anticonvulsant	Yes
Gabapentin	Anticonvulsant	Yes
Lamotrigine	Anticonvulsant	Yes
Levetiracetam	Anticonvulsant	Yes
Oxcarbazepine	Anticonvulsant	Yes
Pregabalin	Anticonvulsant	Yes
Tiagabine	Anticonvulsant	Yes
Topiramate	Anticonvulsant	Yes
Zonisamide	Anticonvulsant	Yes

© 2021 Bello NA et al. *JAMA Network Open.*

ICD-9 **codes for exclusion criteria**

DX	ICD9_definition	Exclusion Condition
392.0	Rheumatic chorea with heart involvement	Heart disease
394.0, 394.1, 394.9	Mitral valve diseases	Heart disease
396.3	Mitral valve insufficiency and aortic valve insufficiency	Heart disease
397.0, 397.1	Diseases of tricuspid valve	Heart disease
398.90, 398.91	"Rheumatic heart disease	Heart disease
410.xx	Acute myocardial infarction	Heart disease
411.1, 411.81, 411.89	Intermediate coronary syndrome	Heart disease
412	Old myocardial infarction	Heart disease
413.1, 413.9	Prinzmetal angina	Heart disease
414.00, 414.01, 414.10, 414.11, 414.12, 414.18	Coronary atherosclerosis, aneurysm and other forms of ischemic heart disease	Heart disease
416.0	Primary pulmonary hypertension	Heart disease
416.2	Chronic pulmonary embolism	Heart disease
416.8	Other chronic pulmonary heart diseases	Heart disease
423.3	Cardiac tamponade	Heart disease
423.9	Unspecified disease of pericardium	Heart disease
424.0, 424.1, 424.2, 424.3	Heart valve disorders	Heart disease
425.11, 425.4	Hypertrophic cardiomyopathies	Heart disease
426.0, 426.10, 426.13, 426.3, 426.4, 426.53, 426.7, 426.81, 426.82, 426.9	Cardiac Conduction Disorders	Heart disease
427.0, 427.1, 427.2	Supraventricular tachycardia	Heart disease
427.31, 427.32	Atrial fibrillation & flutter	Heart disease
427.5	Cardiac arrest	Heart disease
427.81, 427.89, 427.9	Other specified cardiac dysrhythmias	Heart disease
428.0, 428.1, 428.20, 428.21, 428.23, 428.30. 428.31, 428.33, 428.9	Heart failure	Heart disease
429.1, 429.2, 429.3, 429.4, 429.89, 429.9	Heart disease	Heart disease
648.51, 648.53	"Congenital cardiovascular disorders of mother	Heart disease
648.60, 648.61, 648.62, 648.63, 648.64	Other cardiovascular diseases of mother,	Heart disease
250.40, 250.41, 250.42, 250.43	"Diabetes with renal manifestations	Kidney disease
581.1, 581.3, 581.81, 581.89, 581.9	Nephrotic syndrome	Kidney disease
582.0, 582.1, 582.2, 582.4, 582.81, 582.89, 582.9	Chronic glomerulonephritis	Kidney disease
583.0, 583.1, 583.2, 583.81, 583.89, 583.9	Nephritis and nephropathy	Kidney disease

© 2021 Bello NA et al. *JAMA Network Open.*

DX	ICD9_definition	Exclusion Condition
585, 585.1, 585.2, 585.3, 585.4, 585.5, 585.6, 585.9	Chronic kidney disease	Kidney disease
586	"Renal failure	Kidney disease
587	"Renal sclerosis	Kidney disease
588.8, 588.81, 588.89, 588.9	Disorders resulting from renal impairment	Kidney disease
589.0, 589.9	Small kidney	Kidney disease
646.20, 646.21, 646.23	Unspecified renal disease in pregnancy	Kidney disease
791.0	Proteinuria	Kidney disease
V45.11	Renal dialysis status	Kidney disease
V56.x	Dialysis	Kidney disease
282.60, 282.61, 282.62, 282.63, 282.64, 282.68, 282.69	"Sickle-cell disease	Sickle cell anemia
710.0	Systemic lupus erythematosus	Systemic lupus erythematosus

© 2021 Bello NA et al. *JAMA Network Open.*

eTable 2. *ICD-9* Codes for Hypertension

**Hypertension Complicating Pregnancy, Childbirth and Puerperium***		
ICD9 Code	Description	Type
642.0x	Benign essential hypertension	Chronic
642.1x	Hypertension secondary to renal disease	Chronic
642.2x	Other pre-existing hypertension	Chronic
642.3x	Transient hypertension of pregnancy (gestational hypertension)	Gestational
642.7x	Eclampsia, preeclampsia superimposed on pre-existing hypertension	Chronic & Preeclampsia
642.9x	Unspecified hypertension of pregnancy	Chronic
	* Requires a fifth digit<br>0 – unspecified episode of care<br>1 – with or without mention of antepartum condition<br>2 – postpartum complication<br>3 – antepartum complication<br>4 – postpartum complication	
**Circulatory System – Hypertensive Diseases**		
401	Essential hypertension	Chronic
401.0	Malignant essential hypertension	Chronic
401.1	Benign essential hypertension	Chronic
401.9	Unspecified essential hypertension	Chronic
402	Hypertensive Heart Disease	Chronic
402.0	Malignant hypertensive heart disease	Chronic
402.00	Malignant hypertensive heart disease – without heart failure	Chronic
402.01	Malignant hypertensive heart disease – with heart failure	Chronic
402.1	Benign hypertensive heart disease	Chronic
402.10	Benign hypertensive heart disease – without heart failure	Chronic
402.11	Benign hypertensive heart disease – with heart failure	Chronic
403	Hypertensive chronic kidney disease**	Chronic
403.0x	Malignant hypertensive chronic kidney disease	Chronic
403.1x	Benign hypertensive chronic kidney disease	Chronic
403.9x	Unspecified hypertensive chronic kidney disease	Chronic
	** A fifth digit may be added for sub-classification of 403<br>0 – with chronic kidney disease stage I through stage IV<br>1 – with chronic kidney disease stage V or end stage renal disease	
404	Hypertensive heart and chronic kidney disease***	Chronic
404.0x	Malignant hypertensive heart and chronic kidney disease	Chronic
404.1x	Benign hypertensive heart and chronic kidney disease	Chronic
404.9x	Unspecified hypertensive heart and chronic kidney disease	Chronic
	*** A fifth digit may be added for sub-classification of 404<br>0 – without heart failure and with chronic kidney disease stage I to IV<br>1 – with heart failure and with chronic kidney disease stage I to IV<br>2 - without heart failure and with chronic kidney disease stage V or end stage	
405	Secondary hypertension	Chronic
405.0	Malignant secondary hypertension	Chronic
405.01	Malignant renovascular secondary hypertension	Chronic
405.09	Other malignant secondary hypertension	Chronic
405.1	Benign secondary hypertension	Chronic
405.11	Benign renovascular secondary hypertension	Chronic
405.19	Other benign secondary hypertension	Chronic
405.9	Unspecified secondary hypertension	Chronic

© 2021 Bello NA et al. *JAMA Network Open.*

405.91	Unspecified renovascular secondary hypertension	Chronic
405.99	Other unspecified renovascular secondary hypertension	Chronic
**Preeclampsia & Eclampsia***		
642.4	Mild or unspecified preeclampsia	Preeclampsia
642.5	Severe preeclampsia	Preeclampsia
642.6	Eclampsia	Eclampsia
642.7x	Preeclampsia or eclampsia superimposed on pre-existing hypertension	Preeclampsia
	* Requires a fifth digit<br>0 – unspecified episode of care<br>1 – with or without mention of antepartum condition<br>2 – postpartum complication<br>3 – antepartum complication<br>4 – postpartum complication	

© 2021 Bello NA et al. *JAMA Network Open.*

eFigure 1. Cohort Inclusion/Exclusion Flow Diagram

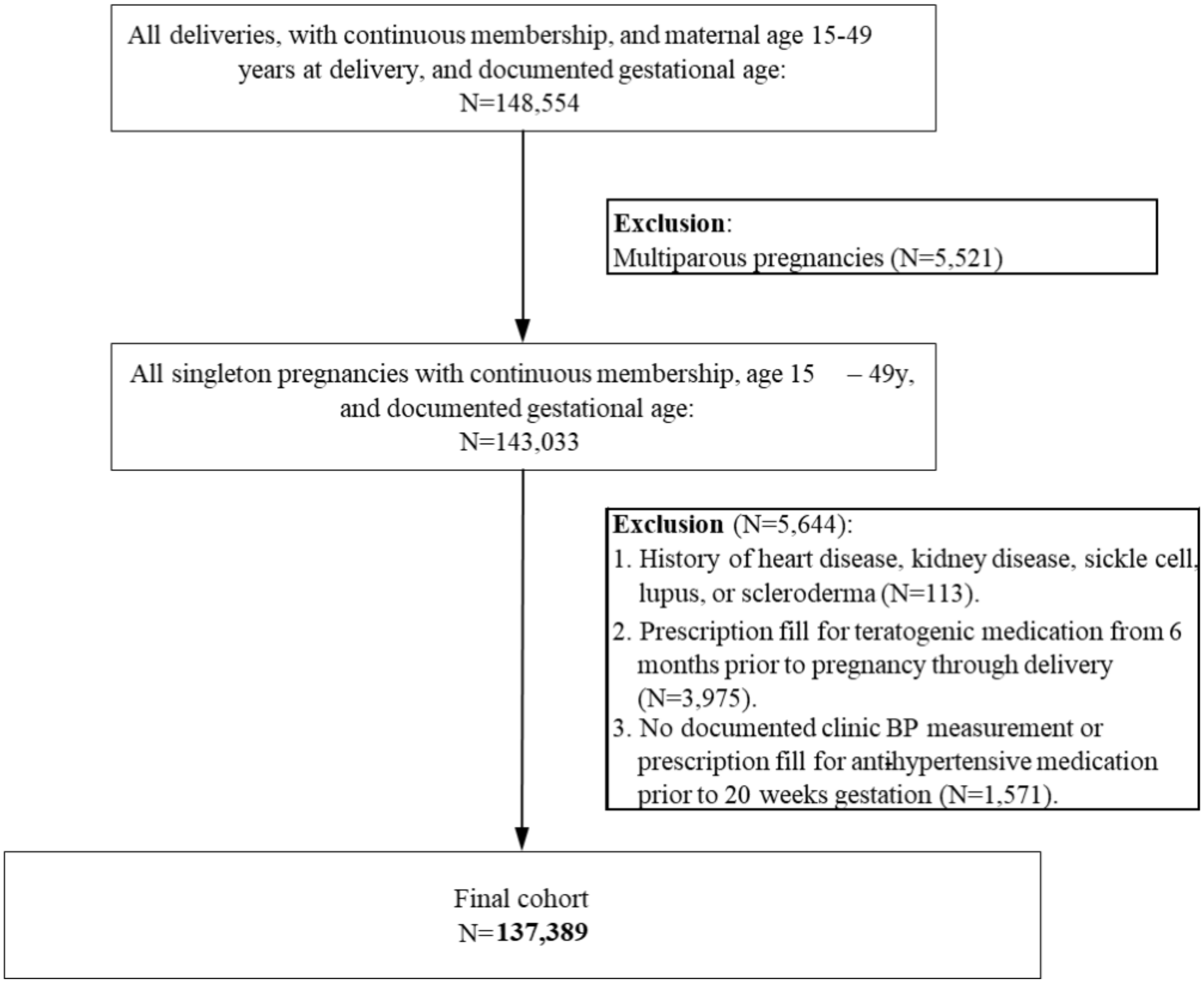

© 2021 Bello NA et al. *JAMA Network Open.*

eFigure 2. Fetal/Neonatal Outcomes: Preterm Birth, Small for Gestational Age (SGA), Neonatal Intensive Care Unit (NICU) Admission.

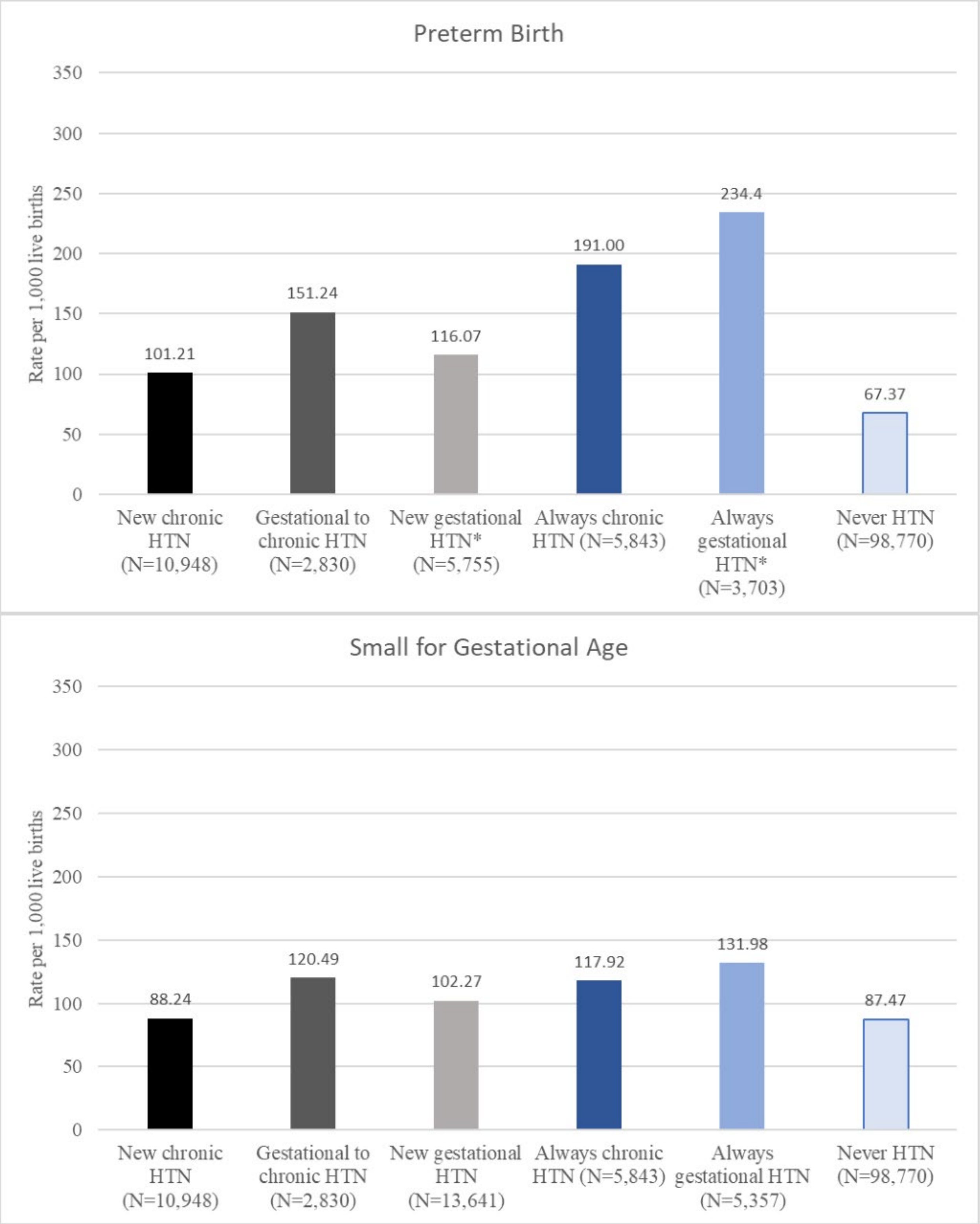

© 2021 Bello NA et al. *JAMA Network Open.*

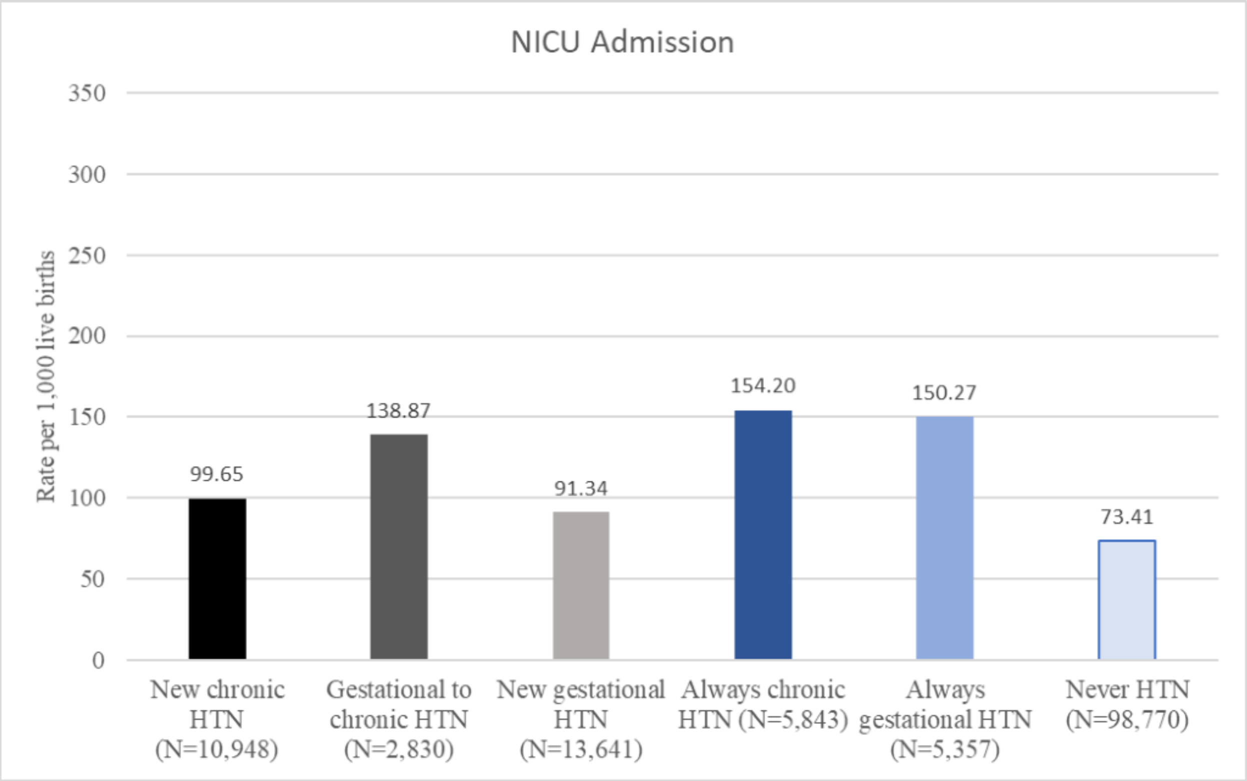

Abbreviations: BP, blood pressure; HTN= hypertension; NICU, neonatal intensive care unit

* For analyses involving preterm birth, new gestational hypertension N=5,755 and always gestational hypertension N=3,703. Women who were diagnosed with gestational hypertension after 37 weeks were excluded from these analyses.

© 2021 Bello NA et al. *JAMA Network Open.*
